# The Use of Quasi-Isothermal Modulated Temperature Differential Scanning Calorimetry for the Characterization of Slow Crystallization Processes in Lipid-Based Solid Self-Emulsifying Systems

**DOI:** 10.1007/s11095-014-1535-8

**Published:** 2014-10-18

**Authors:** Sarah O. Otun, Elizabeth Meehan, Sheng Qi, Duncan Q. M. Craig

**Affiliations:** 1 School of Pharmacy, University of East Anglia, Earlham Road, Norwich, Norfolk NR4 7TJ UK; 2 Pharmaceutical Development, Medicines Development, AstraZeneca, Silk Road Business Park, Charterway, Macclesfield, Cheshire SK10 2NA UK; 3 University College London School of Pharmacy, 29-39 Brunswick Square, London, WC1N 1AX UK

**Keywords:** crystallization, Gelucire 44/14, lipid, Quasi-Isothermal MTDSC, thermal analysis

## Abstract

**Purpose:**

Slow or incomplete crystallization may be a significant manufacturing issue for solid lipid-based dosage forms, yet little information is available on this phenomenon. In this investigation we suggest a novel means by which slow solidification may be monitored in Gelucire 44/14 using quasi-isothermal modulated temperature DSC (QiMTDSC).

**Methods:**

Conventional linear heating and cooling DSC methods were employed, along with hot stage microscopy (HSM), for basic thermal profiling of Gelucire 44/14. QiMTDSC experiments were performed on cooling from the melt, using a range of incremental decreases in temperature and isothermal measurement periods.

**Results:**

DSC and HSM highlighted the main (primary) crystallization transition; solid fat content analysis and kinetic analysis were used to profile the solidification process. The heat capacity profile from QiMTDSC indicated that after an initial energetic primary crystallisation, the lipid underwent a slower period of crystallization which continued to manifest at much lower temperatures than indicated by standard DSC.

**Conclusions:**

We present evidence that Gelucire 44/14 undergoes an initial crystallization followed by a secondary, slower process. QIMTDSC appears to be a promising tool in the investigation of this secondary crystallization process.

## Introduction

There has been a long-standing interest in the use of lipid excipients for drug delivery, particularly for bioavailability enhancement of poorly soluble drugs. More specifically, lipids have been shown to have application as solvents and solubilising agents, as means of enhancing lymphatic transport, for modulation of enterocyte-based drug transport and disposition, as sustained release agents and as coating materials for either taste masking or protection of drugs (1). They have also been found to have the capability of enhancing and standardising drug absorption across the gastrointestinal tract which can be advantageous for the formulation of drug compounds with a low therapeutic index (2). Lipids, which include fatty acids and their derivatives (particularly glycerides), can be further classified into oils, waxes, fats, and more complex lipids including phospholipids and lipoproteins which have direct involvement in biological processes. The choice of lipid for the enhancement of oral bioavailability does, however, tend to be dominated by vegetable or dietary oils and their derivatives (1).

A survey carried out in the UK reported that lipid-based formulations account for approximately 2% of commercially available drug products (3). While this represents a significant market share, these systems are recognized as involving concomitant challenges for manufacture, as the mechanical properties of both solid and liquid lipids require the use of more specialised processing techniques. Issues include the complexity of the chemical and physical structure, stability issues, difficulties associated with scale up, limited solubility of some drugs within lipid carriers, pre-absorptive gastrointestinal processing and a lack of full understanding regarding *in vivo* behaviour and *in vitro*/*in vivo* correlation (4). Nevertheless, the field continues to advance, particularly with the continued introduction of ‘difficult’ drugs into the development process that require non-conventional formulation approaches.

One of the most widely studied solid lipid excipients is Gelucire 44/14, this being an inert, amphiphilic excipient which belongs to the lauryl macroglyceride group of compounds. The dual numbering system refers to the approximate melting point and HLB value of the lipid respectively. This material consists of a combination of mono- and di-esters of polyethylene glycol (PEG) with fatty acids and glycerides, the most common constituent fatty acid being lauric acid, along with free PEGs and glycerol. Gelucire 44/14 displays a number of interesting properties, particularly on contact with water whereby, unlike the majority of solid lipid systems, it emulsifies to form a microfine dispersion of oil particles (5). This process is believed to be attributable to the compositional balance of components, whereby the mono- and di-esters of PEG act as surfactants, the monoglycerides act as cosurfactants, while the di- and triglycerides comprise the oily phase (6), although why this material behaves in this manner when compositionally similar materials simply slowly erode on contact with water is not yet clear. Gelucire 44/14 is also thought to have the capability of forming micelles (7, 8), with Abdul-Fattah and Bhargava (9) arguing that it should be considered to be a non-ionic surfactant. Pharmaceutical products incorporating Gelucire 44/14 include Solufen^R^, Lipofen^R^ and Fenofibrate Winthrop^R^.

Gelucire 44/14 lends itself to formulation into solid self-emulsifying drug delivery systems (solid SEDDS), potentially as the sole excipient, using techniques such as liquid (melt) filling into hard gelatin capsules. This approach, as well as increasing bioavailability of insoluble drugs, can also enhance stomach tolerance of irritant drugs (6). The manufacture of Gelucire formulations typically involves transformation into the fully liquid state before undergoing cooling and crystallization. However, the complexity of the chemical and physical structure and behaviour of multicomponent lipids results in the strong potential for structural variability depending on the manufacturing process (10). Indeed, pure triglycerides display a highly complex polymorphic profile, while materials composed of several lipids may exhibit not only polymorphism but also the formation of a wide array of mixed crystals, rendering characterisation difficult (11, 12). A further issue, which has been identified at the level of manufacture but has not been the subject of extensive academic study within the pharmaceutical arena, is the crystallization process itself in terms of secondary ‘hardening’ whereby the physical properties of the lipid alter on further cooling or storage after the main crystallization process has taken place. Such effects are well recognized in the food science literature, being particularly associated with rapid crystallization whereby the equilibrium microstructure does not fully form due to mass transfer and other effects. For example, for blends of palm oil and palm olein it was suggested that the higher melting triacylglycerols crystallize to form nuclei which then act as nuclei for crystallization of the lower melting species. However, this secondary crystallization may be complicated, and hence slowed, by the possibility of those lower melting components solubilising rather than growing onto the nuclei (13). Similarly, Smith *et al.* (14) studied the cooling of saturated solutions of tripalmitoylglycerol in medium chain triglycerides using NMR and noted a two- step process. The first was rapid, taking place over a few minutes, and accounted for more than two-thirds of the total recrystallization while the second step was considerably slower, taking place over a timescale of hours to days. The authors ascribed these two steps to growth on pitted (rapid) and smooth (slow) crystal faces on the tripalmitoylglycerol following partial dissolution during heating; this may or may not be applicable to the current system whereby liquifaction is effectively complete prior to cooling, but nevertheless represents an interesting possible mechanism for consideration later.

In this study, we examine both the main and secondary crystallization of Gelucire 44/14 using a range of thermal and visual methods. In particular we introduce the use of Quasi-isothermal Modulated Temperature Differential Scanning Calorimetry (QiMTDSC) as a novel means of detecting these secondary crystallization processes. More specifically, the basic expression for conventional MTDSC is given by

1 $$ T={T}_o+\beta t+{A}_T \sin \left(\omega t-\theta \right) $$

where *T* is the temperature at time *t* and *T*_*0*_ the initial temperature, *β* is the underlying heating rate, *A*_*T*_ is the amplitude of the temperature modulation with angular frequency *ω* and *θ* is the phase shift with regard to the reference pan. The total heat flow response may be deconvoluted into the reversing and non-reversing heat capacity. The former, *C*_*p,rev*_, is calculated from the amplitude of the first harmonic of the heat flow *A*_*P*_, such that for a sample of mass *m*

2 $$ {C}_{p, rev}=\frac{A_P}{m{A}_{T*}} $$

where *A*_T*_ = *ωA*_*T*_. The non-reversing heat capacity signal is simply the difference between the total and reversing signals at any given temperature. More details of the deconvolution process may be found in a number of references (e.g. 15, 16). The advantage of the modulated technique is that the sensitivity of the heat capacity measurement is enhanced considerably due to the use of the modulated signal, thereby facilitating measurement of events such as glass transitions which are essentially heat capacity changes. As the heat capacity is a fundamental measure of molecular mobility, measurement of this parameter may also be used to detect changes in the physical characteristics of a sample under study.

QiMTDSC is a variant of traditional MTDSC which involves the holding and modulation of a sample at a specific temperature for extended periods of time. The temperature can be incrementally increased or decreased through a transition, reducing or effectively eliminating the influence of scanning rate, while also allowing investigation of time dependent processes. The quasi-isothermal method effectively uses a heating rate of zero, hence the heat capacity is measured as a function of time at any particular temperature. Outside the glass transition region (which is not applicable in the present case), the thermodynamic heat capacity reaches equilibrium very quickly (17), while latent heat effects will be seen in the non-reversing signal and may thus be isolated. Consequently, by measuring the change in heat capacity one may observe changes in physical state as a function of time. A further advantage is that for very slow processes the enthalpic response may be so small over the timescale of an experiment that it becomes extremely difficult to detect, hence the monitoring of heat capacity offers an alternative approach to enthalpy measurements. The crystallization process can also be observed through the use of Lissajous figures (17), whereby the modulated heat flow is plotted against modulated temperature. This allows observation of the reproducibility of the sine wave heat flow modulations between consecutive isothermal modulation periods. In this manner, therefore, it is intended that the slow and energetically subtle secondary crystallization process of Gelucire 44/14 may be detected using the quasi-isothermal technique.

## Materials and Methods

### Materials

Gelucire 44/14 (Lot Number 103201) was kindly provided by Gattefossé (Lyon, France) and used as received.

### Conventional Differential Scanning Calorimetry

Conventional DSC experiments were performed under a dry nitrogen environment, with a purge rate of 50 ml/min. Calibration of the instrument was conducted prior to experimentation, involving cell resistance and capacitance calibrations, cell constant calibrations using indium standard, and finally temperature calibrations using benzoic acid and n-octadecane. Temperature calibrations were carried out at the same rate as intended for sample analysis. Experiments were conducted in crimped TA standard aluminium pans, all of a similar weight, with a sample weight range of 2 to 2.5 mg taken directly from the original container. Samples were heated from 0 to 60 at 10°C/min, held isothermally for 10 min and then cooled at a rate of 0.5, 2, 10 or 20°C/min. Experiments were run four times. Solid fat content diagrams were obtained by calculating the enthalpy of fusion at a series of temperatures through the melting process and expressing those values in terms of percentage of the total enthalpy as a function of temperature.

### Quasi-Isothermal Modulated Temperature Differential Scanning Calorimetry

Quasi-isothermal Modulated Temperature DSC (QMTDSC) experiments were performed under a dry nitrogen environment at a purge rate of 50 ml/min. Calibration of the instrument was conducted, prior to experimentation, as per conventional DSC. An additional heat capacity calibration for MTDSC was also carried out using aluminium oxide. Samples in the weight range 2 to 2.5 mg were prepared into TA standard aluminium pans or Tzero aluminium pans, all of similar weight. All samples were heated above the melting temperature at 10°C/min to 60°C, held for 10 min and then cooled to the point of QiMTDSC. All methods employed an amplitude of ±1°C and a period of 60 s.

Method One Cooled from 40 to −10°C in 5°C increments, with an isotherm of 60 min at each increment.
Method Two Cooled from 35 to 5°C in 1°C increments, with an isotherm of 20 min at each increment.
Method Three Held isothermally for 720 min (12 h) at 29, 30, 31, 32, 33, 35 or 40°C. Sample also held at 29°C for 48 h.

### Hot Stage Microscopy

Samples for analysis were applied to glass microscope slides and heated from 30 to 50°C at 10°C/min, cooled to room temperature, then re-heated to 50°C. Images were captured at ×20 magnification, under polarised light. It should be noted that the apparatus was not capable of controlling the rate at which samples under investigation could be cooled, however the rate of cooling was calculated to be approximately 2°C/min.

## Results

### Melting behaviour using conventional DSC

**Fig. 1:**
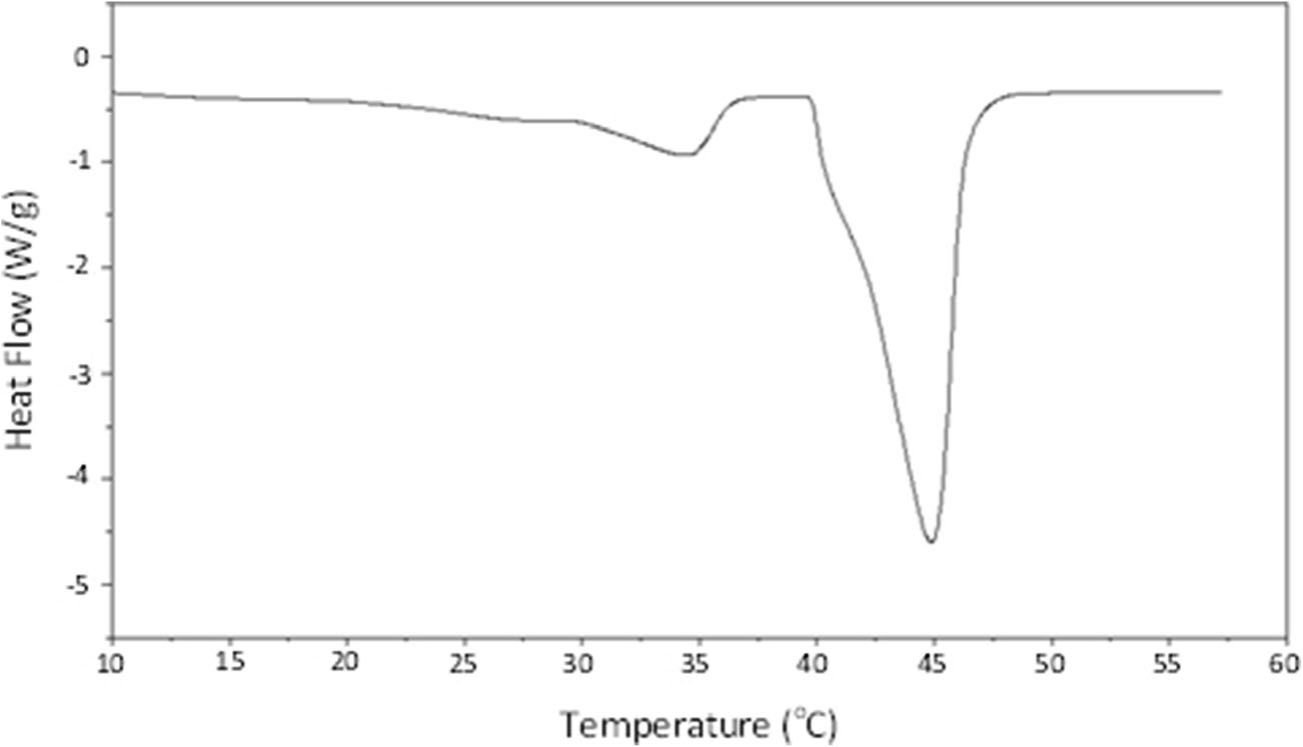
Heat flow response against temperature of Gelucire 44/14 upon heating at 10°C/min.

Upon application of a linear heating signal, Gelucire 44/14 was observed to exhibit a characteristic double melting endotherm (5), composed of a small leading peak with an extrapolated melting onset temperature (Tm_(onset)_) of 28.3°C and a primary melt occurring at 39.9°C (Fig. 1). The leading peak appeared to consist of two components, thought to reflect the lower melting point fractions of the lipid (18). The larger primary melt endotherm also demonstrated a slight shoulder. However, due to the multiple components of the lipid and their complex interaction, which are not yet fully understood, the endotherms cannot be assigned to any specific components with confidence (19). One notable exception to this for the Gelucires lies in the study of Olivon *et al.* (20) whereby temperature dependent small angle XRD was used to identify the species responsible for the melting peaks of Gelucire 50/13.

### Crystallisation

There are some particular considerations pertinent to using thermal methods for measuring crystallization. Melting, as a first order thermodynamic process, is not affected by increasing heating rate. Crystallisation, however, is kinetically controlled and therefore the temperature at which it occurs is reduced with increasing rate of cooling. The crystallisation onset temperature (Tc_(onset)_), found by extrapolation of the main lead curve to the baseline, reduces in temperature from 28.8°C ± 0.9 (Tc_(max)_ 27.4°C ± 0.9; ΔH 98.7 J/g ± 8.4) cooling at 0.5°C/min, to 20.0°C ± 2.3 (Tc_(max)_ 12.5°C ± 3.0; ΔH 100.6 J/g ± 1.8) at 20°C/min.

**Fig. 2:**
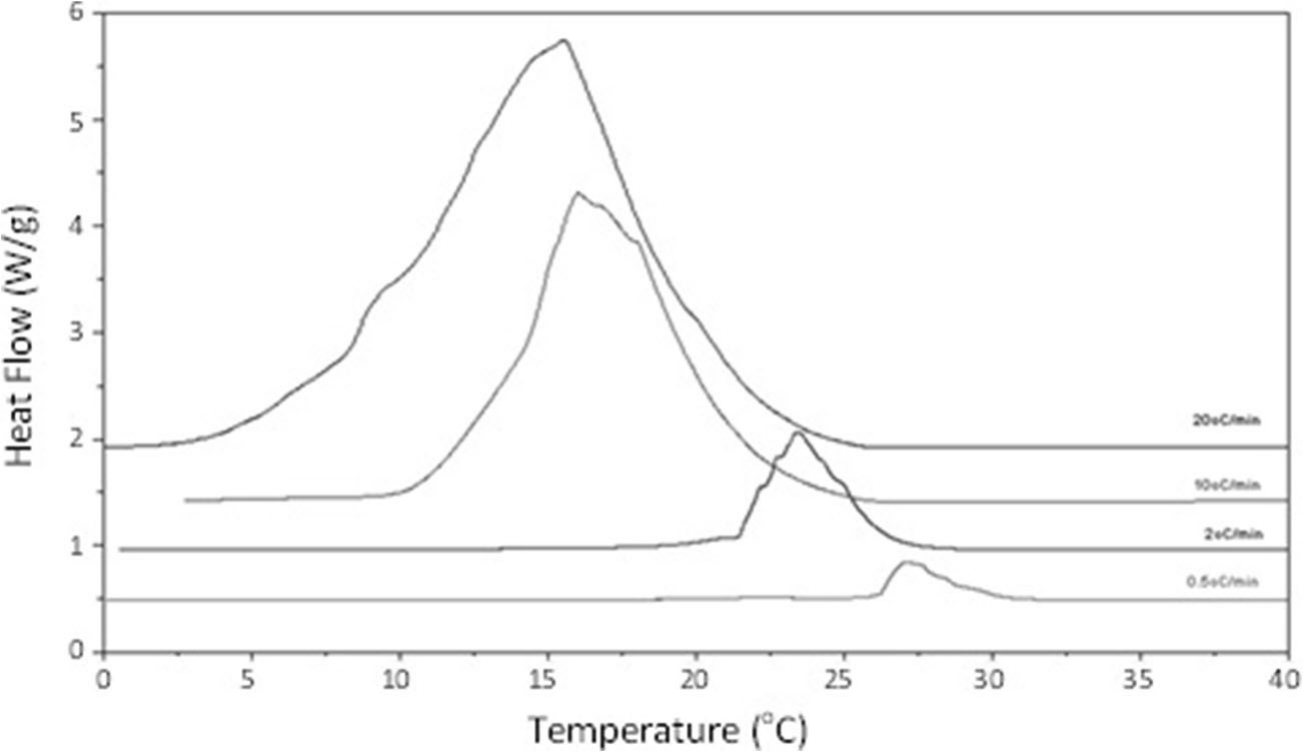
Heat flow against temperature signal of Gelucire 44/14 upon cooling at varying rates.

Over and above this effect, modification of lipid mechanical properties upon variation of cooling rate due to the formation of mixed glyceride crystals is well known (e.g. 24). Increasing the cooling rate also enhances sensitivity, making transitions appear larger when expressed as heat flow due to an increase in energy flow per unit time. This is demonstrated by Fig. 2 in which the crystallisation exotherm increases considerably in size (in terms of heat flow) at higher rates.

As mentioned previously, the effects of cooling conditions has been widely studied in the lipid field, with slow cooling rates generally resulting in larger mesophase crystal growth and the generation of more stable polymorphs (24). For Gelucires, however, faster cooling resulted in the formation of a homogeneous system while a slower rate encourages fractionation of the various lipid components into different microscopic regions (19).

### Crystallisation Kinetics

Despite the complexity of the solidification process, crystal growth in lipids is routinely described by the well-known Avrami approach (21, 22) which takes the form:

3 $$ \left(1-X\right)= exp\left(-k{t}^n\right) $$

where *X* is the crystal fraction at time *t*, *k* is a crystallisation rate constant and *n* is the Avrami exponent, which may in turn be used to estimate the mechanism and processes associated with crystallization. The value of *n* can be expressed as

4 $$ n={n}_d+{n}_n $$

where *n*_*d*_ relates to the dimensionality of crystal growth, and *n*_*n*_ is the time dependence of nucleation. *n*_*d*_ can be calculated to the value of 1, 2 or 3 corresponding to one dimensional growth, two dimensional lamellar aggregates (axialites) or three dimensional aggregate superstructures of radial lamellae (spherulites) respectively. The value of *n*_*n*_ can assume the integers 0 or 1 signifying instantaneous nucleation or spontaneous nucleation respectively (23).

**Fig. 3:**
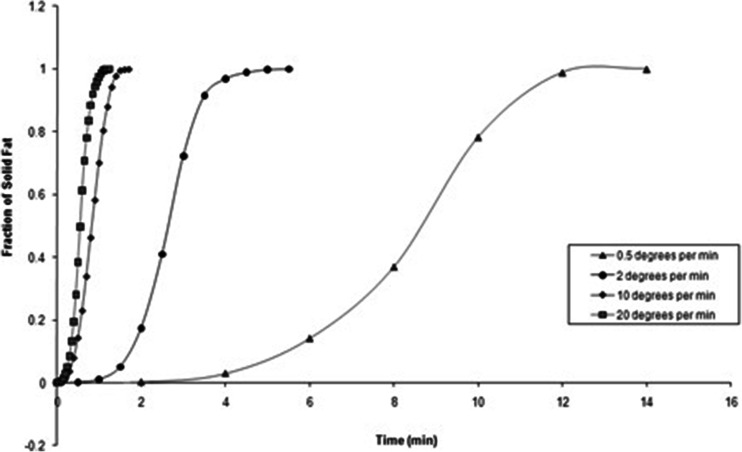
Fraction of solid fat of Gelucire 44/14 *versus* time during crystallisation upon cooling at varying rates.

A further simple and useful means of monitoring the solidifcation process is the use of solid fat content analysis, whereby the proportionate completion of the crystallization exotherm is plotted as a function of temperature or time at a known cooling rate (24), as shown in Fig. 3.

Using the solid fat fraction at time *t* against time data (Fig. 3) it was possible to perform Avrami analysis. The Avrami modelling parameters are illustrated in Table 1. The data was found to fit well, with R^2^ values between 0.9934 and 0.9998. The crystallisation rate constant, k, was observed to increase with increasing cooling rate, as is typical for lipids (24).

**Table 1:** Avrami modelling parameters for the solid fat data of Gelucire 44/14

Cooling rate(°C/min)	*n*	k (min^-n^)	R^2^
0.5	4	0.0001	0.9934
2	4	0.0015	0.9981
10	3.05	1.22	0.9998
20	3.24	4.64	0.9991

The value of the calculated *n* parameter gave an indication as to the time dependence of nucleation and also the dimensionality of the crystal growth. Slower cooling rates illustrated an *n* value of 4 suggesting heterogeneous nucleation and spherulitic growth from sporadically formed nuclei. This also indicates that the rate of nucleation was constant and independent of time. Cooling rates of 10 and 20°C/min demonstrated an *n* value of 3 (to the nearest integer), suggesting a shift in mechanism which may reflect spherulitic growth from instantaneous nuclei.

### Quasi-isothermal MTDSC

As outlined previously, quasi-isothermal MTDSC allows the investigation of kinetic processes such as crystallisation at a range of single temperatures, thereby not only allowing the monitoring the process in real time but also, in theory, allowing identification of the critical temperatures at which these processes commence and are complete. Here we use the technique primarily for identification of processes rather than quantitative kinetic analysis as there remain uncertainties regarding the effects of equilibration at each temperature, hence more work is required to eliminate these effects before such analysis may be reliably performed. Nevertheless, we seek to establish proof of concept that the method may indeed be used to identify and potentially analyse slow crystallisation processes, as well as to study the effects of drug incorporation on these secondary processes.

**Fig. 4:**
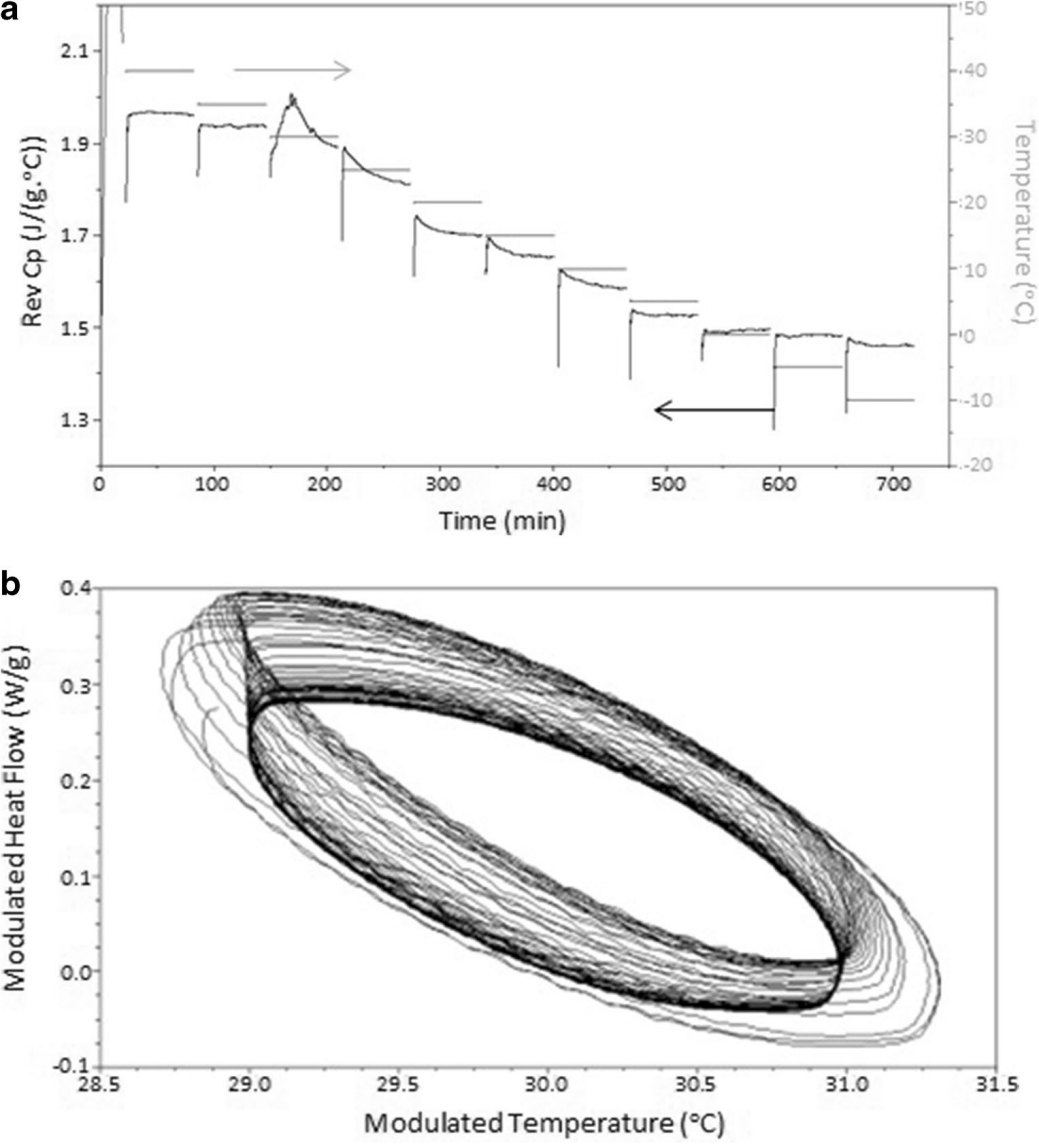
(**a**) Reversing heat capacity *versus* time signal for Gelucire 44/14 QiMTDSC 60 min isotherm on cooling with 5°C increments (Arrows indicate the scale corresponding to the two data sets). (**b**) Lissajous plot (modulated heat flow against modulated temperature) of the sine wave heat flow modulations at 30°C. The process begins with the less dense ellipses and terminates in the dense region, indicating equilibration.

After analysis of Gelucire 44/14 using *Method One*, which involved cooling from 40 to −10°C in 5°C increments with an isotherm of 60 min at each increment, crystallisation appeared to occur during the 30°C isothermal period. This was indicated by the abrupt increase in reversing heat capacity and also the deviation of the sine wave modulations from steady state in the Lissajous analysis (Fig. 4). This method of data presentation is essentially a means of presenting multiple sinusoidal signals against each other. These can be given simply by

5 $$ x=A\kern0.5em  sin\left( at+\delta \right) $$

and

6 $$ y= Bsin(bt) $$

where A and B are the amplitudes, δ is the phase difference and a and b are the frequencies. If a = b it means that the input and output, or stimulus and response, are both sinusoidal with the same frequency, in which case the curve takes the form of a closed ellipse. By plotting the modulated heat flow against modulated temperature, each cycle of the response is presented as a superimposing ellipse, in turn allowing both the steady state nature (or otherwise) of the response to be determined in terms of the superimposibility of the response as well as the detection of non-linear responses such as crystallisation via distortion of the ellipse. It should be noted that the major axis of the ellipse represents the heat capacity of the sample, hence a change in orientation with time also indicates a change in C_p_.

In the current case, deviation of the Lissajous plot from the steady state was at its maximum during the first 31 min of the 60 min modulation at 30°C; after this period the sine wave curves reached equilibrium (see Fig. 4b). Close inspection of the figure indicates that the main axis, which reflects the heat capacity of the system, shifts upwards and then down after approximately 12 cycles (12 min); we believe that this may reflect the exothermic effects associated with the solidification process which may take the system out of steady state while temperature equilibration is established, thus complicating quantitative analysis of the data.

These data therefore implied that the *T*_*c*_ of Gelucire 44/14, independent of cooling rate, is likely to lie between 30 and 35°C. The exact temperature could not however be determined from this analysis as the temperature increment was too large. A further observation from Fig. 4a, also reflected in the Lissajous figures at the corresponding temperatures (data not shown) changes in reversing heat flow (and hence heat capacity) were still observable as a function of time, even at temperatures well below the main crystallization event. This implies that even though the main crystallization event had taken place between 30 and 35°C, the process was continuing at lower temperatures, in turn indicating secondary, slow crystallization processes.

**Fig. 5:**
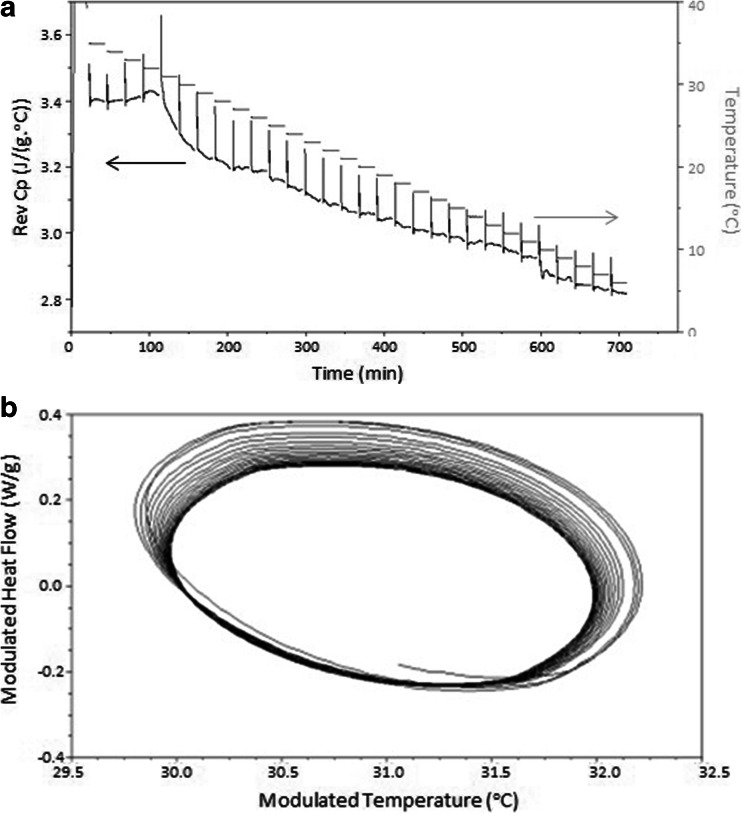
(**a**) Reversing heat capacity *versus* time signal for Gelucire 44/14 QIMTDSC 20 min isotherm on cooling with 1°C increments (Arrows indicate the scale corresponding to the two data sets). (**b**) Lissajous plot (modulated heat flow against modulated temperature) of the sine wave heat flow modulations at 31°C.

*Method 2* subjected Gelucire 44/14 samples to a reduced temperature increment of 1°C, from 35 to 5°C, for a more accurate determination of the ‘equilibrium’ Tc (Fig. 5a and b).

When held isothermally for 20 min at 1°C increments, deviations from the equilibrium sinusoidal response and a change in shape and size of the sine wave ellipses in the Lissajous analyses were found to commence at 32°C and to be most clearly manifest at 31°C (see Fig. 5b). It was found, however, as before that a secondary, more extended period of slow crystallisation was present after 31°C in the reversing heat capacity signal, continuing until the conclusion of the experiment at 5°C. This is seen from the change in reversing heat flow through each of the incremental periods in Fig. 5a, again indicating that the crystallization process is far from complete after the main exothermic event.

**Fig. 6:**
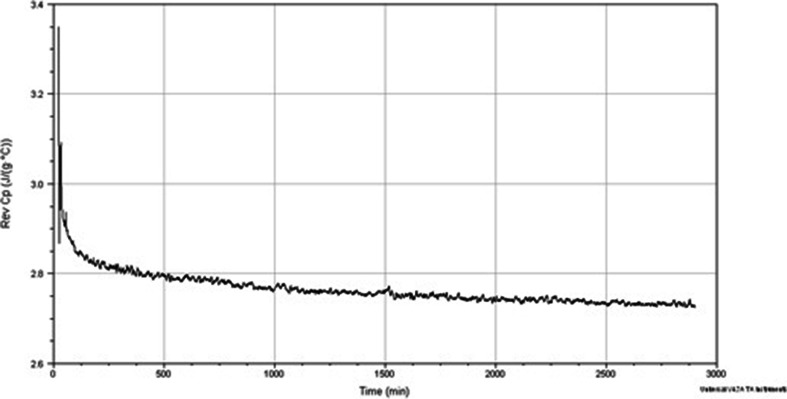
Reversing heat capacity *versus* time signal for Gelucire 44/14 12 h QiMTDSC at 29°C over 48 h

*Method Three* involved holding the sample isothermally for 720 min (12 h) at temperatures from 29 to 40°C, with a further study on the system held at 29°C for an extended period of 48 h. Crystallisation was found to occur when held isothermally for 12 h at 29, 30, 31 and 32°C; above this temperature however, no obvious transition was present in the reversing heat capacity signal over 12 h (data not shown). It was noted that for all samples held at temperatures at or below 32°C, an initial crystallisation response was seen followed by a steady decrease in heat capacity which we suggest corresponds to the secondary process. The trend is demonstrated in Fig. 6 whereby the heat capacity is seen to decrease steadily over a 48 h period.

### Hot Stage Microscopy

**Fig. 7:**
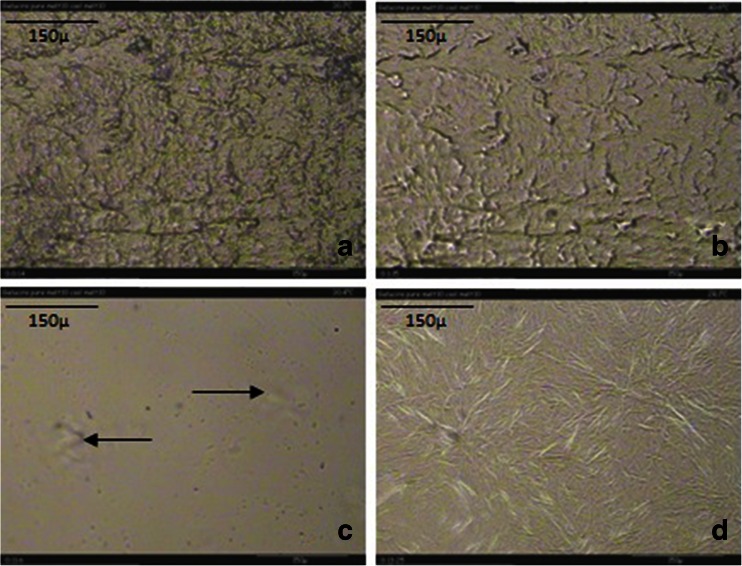
HSM Images of Gelucire 44/14; (**a**) Start (ambient); (**b**) Melting at 36.0°C; (**c**) Nucleation on cooling at 30.4°C; (**d**) Crystal growth at 28.8°C.

HSM was employed to visualise the melting and crystallisation transitions of Gelucire 44/14, previously characterised using conventional and QIMTDSC. Figure 7 shows images of these processes. Melting of Gelucire 44/14 appeared to occur over a wide temperature range, beginning at approximately 36°C until completion at 46°C. This is compatible with the behaviour seen with the DSC studies. Upon cooling, nucleation began at 30°C followed by crystal growth until 28°C. The crystals appeared to be spherulitic and radial in nature, growing outwards in finger-like projections.

Upon re-heating, melting commenced at 39°C and was complete by 46°C. Comparing this to data obtained from conventional and QIMTDSC, it is apparent that neither melting of the lower melting point fractions, or the extended slow crystallisation of Gelucire 44/14 are visible using HSM.

## Discussion

The study has addressed the issue of slow secondary crystallization processes in a commonly used lipid, while also introducing new techniques by which the phenomenon may be studied. Slow crystallization is recognized anecdotally within the pharmaceutical industry, yet few studies have directly investigated the process, not least because of the difficulties associated with reliable measurement. Here we show that the main crystallization process may be effectively characterized using conventional DSC and HSM, particularly using solid fat content analysis to quantify the extent of crystallization for complex lipid samples. Similarly, QiMTDSC allows the operator to measure a crystallisation temperature which is effectively independent of cooling rate. However, the secondary processes are energetically (and visually) subtle but may be detected via the change in heat capacity associated with the enhanced solidification process. In order to make these measurements, it is necessary to measure the sample isothermally as it would be impossible to differentiate such subtle changes in heat capacity from those induced by temperature alterations if a non-isothermal process were to be used. By using QiMTDSC we are able to show changes in heat capacity following crystallization that are entirely consistent with secondary solidification processes, hence we suggest that the technique may be a useful tool in detecting such processes. However, quantification remains a challenge due to a combination of the subtle change from the baseline as well as the need to consider temperature equilibration effects during the exothermic crystallization process. Nevertheless, the study has shown that QiMTDSC has a role in monitoring subtle secondary crystallization processes.

## Conclusion

The study has demonstrated the complexity of the crystallization behaviour of Gelucire 44/14, in particular outlining the challenges associated with assessing both the primary and secondary processes associated with solidification. The main crystallization process may be studied using conventional DSC and HSM, particularly using solid fat content analysis combined with kinetic modelling. However the secondary, slow solidification processes which may occur over several hours may be studied more effectively using QiMTDSC, where the heat capacity is measured as a function of time at any given temperature. Here we show both the potential of the method to detect such changes but also outline the associated limitations, particularly in terms of the need for a suitably flat baseline and the need to develop the approach further for quantitative studies.
